# Enthalpy/Entropy Contributions to Conformational KIEs: Theoretical Predictions and Comparison with Experiment

**DOI:** 10.3390/molecules18022281

**Published:** 2013-02-18

**Authors:** Aaron Fong, Matthew P. Meyer, Daniel J. O’Leary

**Affiliations:** 1 Department of Chemistry, Pomona College, 645 North College Avenue, Claremont, CA 91711, USA; E-Mail: ayf02008@mymail.pomona.edu; 2 Department of Chemistry, University of California, Merced, Atwater, CA 95301, USA

**Keywords:** conformational kinetic isotope effect, steric isotope effect, Bigeleisen-Mayer formalism, enthalpy/entropy contributions to isotope effects

## Abstract

Previous theoretical studies of Mislow’s doubly-bridged biphenyl ketone **1** and dihydrodimethylphenanthrene **2** have determined significant entropic contributions to their normal (**1**) and inverse (**2**) conformational kinetic isotope effects (CKIEs). To broaden our investigation, we have used density functional methods to characterize the potential energy surfaces and vibrational frequencies for ground and transition structures of additional systems with measured CKIEs, including [2.2]-metaparacyclophane-*d* (**3**), 1,1'-binaphthyl (**4**), 2,2'-dibromo-[1,1'-biphenyl]-4,4'-dicarboxylic acid (**5**), and the 2-(*N,N,N*-trimethyl)-2'-(*N,N*-dimethyl)-diaminobiphenyl cation (**6**). We have also computed CKIEs in a number of systems whose experimental CKIEs are unknown. These include analogs of **1** in which the C=O groups have been replaced with CH_2_ (**7**), O (**8**), and S (**9**) atoms and ring-expanded variants of **2** containing CH_2_ (**10**), O (**11**), S (**12**), or C=O (**13**) groups. Vibrational entropy contributes to the CKIEs in all of these systems with the exception of cyclophane **3**, whose isotope effect is predicted to be purely enthalpic in origin and whose Bigeleisen-Mayer ZPE term is equivalent to ΔΔ*H*^‡^. There is variable correspondence between these terms in the other molecules studied, thus identifying additional examples of systems in which the Bigeleisen-Mayer formalism does not correlate with Δ*H*/Δ*S* dissections.

## 1. Introduction

**Figure 1:**
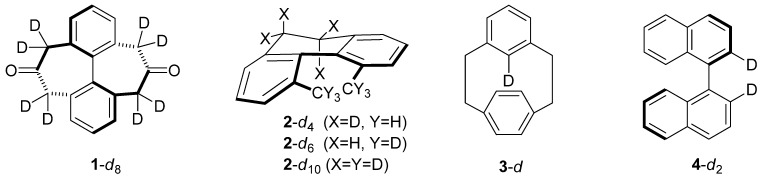
Molecules for which CKIEs have been measured.

Isotope effects are generally thought to be dominated by enthalpic zero-point vibrational energy (ZPE) differences between structures, but it is known that thermal excitation of low vibrational frequencies can produce significant entropic contributions in a number of isotope effects, including conformational kinetic isotope effects (CKIEs) in amide bond rotation [1,2], KIE and equilibrium isotope effects (EIEs) in metal-H_2_ and metal-C-H interactions [3], and EIEs in intramolecular hydrogen bonds [4]. Recently, we reported a computational study of CKIEs in two deuterium-labeled chiral biphenyl systems (**1** and **2**) and found large entropic contributions operative in each [5]. Conformational kinetic isotope effects are manifested in molecular systems whose bond rotation dynamics are affected by the presence of isotopic substitution. Dihydrodimethylphenanthrene **2**-*d*_6_ (Figure 1) is often used as a prototype CKIE example and the isotope effect is usually explained on the basis of the ‘smaller’ –CD_3_ groups—with shorter C−D bonds—more readily slipping past each other and resulting in a faster racemization rate. A steric isotope effect such as this would be expected to be purely enthalpic (ZPE) in origin [6]. But isotopic substitution does not always cause faster conformational processes, and our work confirmed that entropic contributions are responsible for the normal (*k*_H_ > *k*_D_) isotope effect in diketone **1** and serve to diminish a largely enthalpic inverse (*k*_H_ < *k*_D_) isotope effect in phenanthrene **2**.

In diketone **1**-*d*_8_, we discovered the inverse ZPE contribution to the enthalpy (*H*_ZPE_) is masked by an unusually large and normal thermally excited vibrational enthalpy (*H*_vib_) contribution [5]. The consequence of this unique situation is that it nullifies the connectivity normally associated between the Bigeleisen-Mayer [7,8] terms (ZPE, EXC, MMI, and SYM) and enthalpy and entropy. Indeed, the ZPE term is often viewed as the sole contributor to any enthalpic differences in isotopic systems and the EXC/MMI/SYM terms, elaborated upon below, are thought to comprise vibrational and rotational entropy differences. Our study of diketone **1** identified a molecule where this relationship breaks down. This finding is relevant to theory/experiment comparisons, as computed isotope effects are usually dissected into their contributing Bigeleisen-Mayer terms, whereas experimentalists usually measure enthalpic and entropic contributions. 

**Figure 2:**
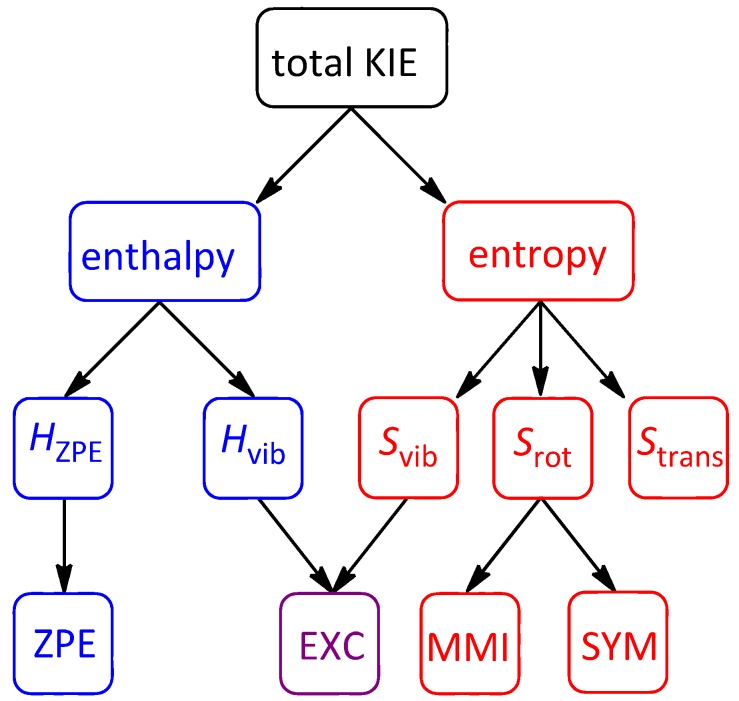
Flowchart illustrating a KIE partitioning into enthalpy and entropy contributions, and their relationship to the Bigeleisen-Mayer ZPE, EXC, MMI, and SYM contributions.

The flow chart shown in Figure 2 illustrates the relationships between the various contributions to isotope effects. As mentioned previously, enthalpy partitions into *H*_ZPE_ and *H*_vib_. The former is identical to the Bigeleisen-Mayer ZPE term while the latter manifests in the EXC term, which accounts for differential thermal excitation of vibrational modes in the structures being compared. Two of the three entropic contributions matter in isotope effects, though for most molecules the vibrational (*S*_vib_) contribution is generally far more important than any rotational (*S*_rot_) contributions. The *S*_vib_ term combines with *H*_vib_ to form the Bigeleisen-Mayer EXC term, while *S*_rot_ within the Bigeleisen-Mayer formalism is further partitioned into a mass-moment of inertia component (MMI) and a symmetry factor (SYM). For the systems being discussed here, the symmetry contributions to *S*_rot_ or SYM tend to involve unitary ratios of rotational symmetry numbers and are thus not important. The same is true of translational entropy (*S*_trans_), which does not change in molecules undergoing conformational rearrangements. Therefore, our discussions of CKIE contributions will tend to focus on enthalpic and entropic contributions (“Δ*H*/Δ*S*”) as compared with the three most significant Bigeleisen-Mayer terms: ZPE, EXC, and MMI.

To provide a sense of what can happen when *H*_vib_ is large and opposite in sign to *H*_ZPE_, the computed Bigeleisen-Mayer terms for the CKIE (1.075, expt: 1.06 [9,10]) in diketone **1**-*d*_8_ at 368 K are: ZPE (1.026). EXC (1.050), and MMI (0.998). The computed enthalpy-entropy contributions in this system are ΔΔ*G*^‡^ (1.075), ΔΔ*H*^‡^ (0.973), and −TΔΔ*S*^‡^ (1.105) [5]. Here, *H*_vib_ is sufficiently large such that the overall ΔΔ*H*^‡^ term is inverse while the ZPE term is normal. The Bigeleisen-Mayer analysis, taken alone, might lead one to conclude that enthalpy and entropy are each contributing a “normal” isotope effect, whereas the Δ*H*/Δ*S* decomposition shows inverse/normal “antagonistic” contributions, where the entropic term is dominant. That deuteration slows the conformational exchange in **1**-*d*_8_ and is due to a vibrationally excited process has been suggested to have implications for cases where deuterium appears to have a larger effective size than protium [11]. It is also worthwhile noting that diketone **1** was among the first of several molecules studied by a number of researchers in the 1960s and 1970s who were interested in understanding steric isotope effects. A steric CKIE was expected to be “inverse” (*k*_D_ > *k*_H_) by virtue of ZPE effects [6]. Researchers had their suspicions that the normal CKIE in **1** was due to entropic factors, but our computational study was the first to find evidence for an entropically-controlled CKIE.

On the other hand, dihydrodimethylphenanthrenes **2**-*d*_4_, **2**-*d*_6_, **2**-*d*_10_ displayed inverse CKIEs and the latter two—whose transition structures were postulated to contain compressed CH_3_/CH_3_ and CD_3_/CD_3_ groups—represented the first experimental examples of a steric isotope effect. But the CKIE in this system is not purely enthalpic in origin, as would be expected for a purely steric isotope effect [12,13]. This was evident when Mislow and co-workers employed variable-temperature experiments and found a large—though not dominant—entropic contribution. Our calculations agreed with the experimental findings and revealed Bigeleisen-Mayer terms for the CKIE (0.888, expt: 0.880 [13]) in **2**-*d*_6_ at 315 K as: ZPE (0.755), EXC (1.182), and MMI (0.995). The computed enthalpy-entropy contributions in this system are ΔΔ*G*^‡^ (0.888), ΔΔ*H*^‡^ (0.743), and −ΔΔ*S*^‡^ (1.193).

This paper reports additional examples of computed CKIEs in medium-sized molecules. Four of the molecules (compounds **3**–**6**) have known CKIEs and thus provide an opportunity for further validation of our theoretical approach as well as offering a retrospective analysis of contributing terms. The remaining compounds (**7**–**13**) have not been studied experimentally, but as structural variants of compounds **1** and **2** they allow us to further probe the origin of the unique CKIEs in these two molecules and perhaps better understand isotope effects generally [14,15,16,17,18].

## 2. Results and Discussion

### 2.1. [2.2]-Metaparacyclophane-d (**3**-d)

[2.2]-Metaparacyclophane-*d* (**3**-*d*, Figure 1) was among the earliest reported examples of a molecule with a steric CKIE. The interconversion process in this molecule requires severe compression of a C-H(D) bond as it passes across the surface of the *p*-disubstituted ring. The CKIE was described by Boekelheide and co-workers in 1972 as “…inverse and its magnitude, *k*_H_/*k*_D_ = 0.833 ± 0.04, is twice as large per deuterium as any other conformational kinetic isotope effect of which we are aware [19,20]”. The enthalpy (Δ*H*^‡^ = 17.0 ± 0.5 kcal/mol) and entropy (Δ*S*^‡^ = −8.8 ± 2.4 cal/mol K) of activation in unlabeled **3** was determined with variable-temperature NMR. Given the uncertainty in determining these parameters, the authors were unable to dissect enthalpic and entropic contributions to the isotope effect.

Our calculations of the CKIE in **3** reproduce the experimental value almost perfectly at the DFT B3LYP/6-31G(d,p) level of theory (0.832, Table 1) using a scaling factor [21] of 0.97. Although we are only reporting the B3LYP/6-31G(d,p) results here, we have tested the reproducibility of our calculations with a number of approaches. Using cyclophane **3 **computed at 308 K as an example, we find: MP2/6-31G(d,p) frequencies scaled by 0.96, KIE = 0.791; DFT B97D/6-31G(d,p) frequencies scaled by 0.97, KIE = 0.830; HF/6-31G(d,p) frequencies scaled by 0.918, KIE = 0.797.

**Figure 3:**
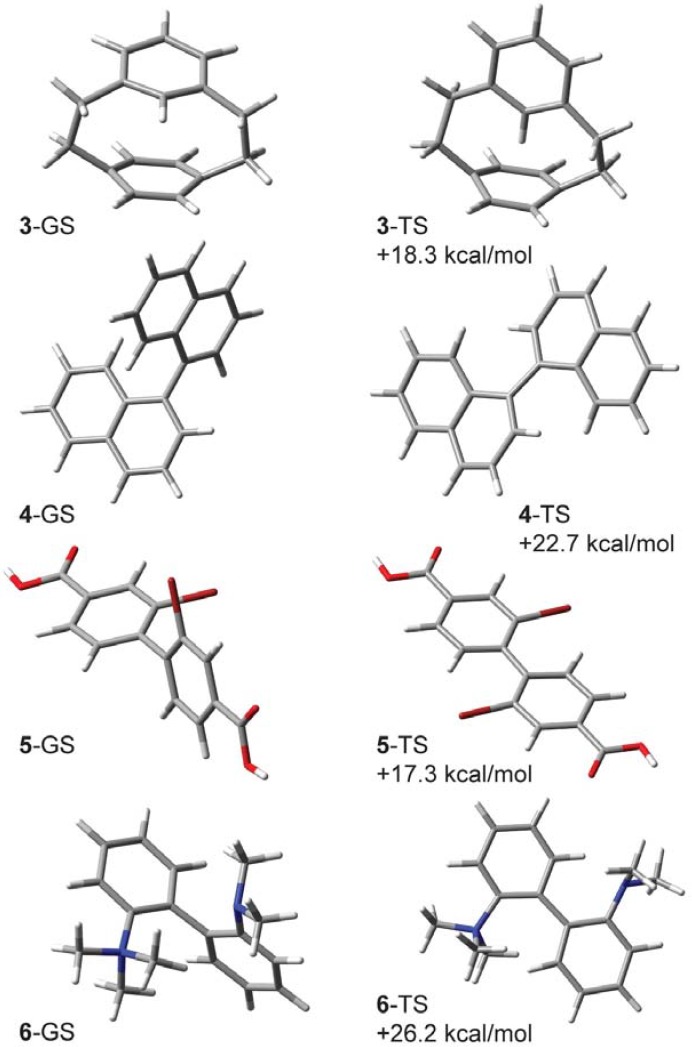
B3LYP/6-31G(d,p) lowest-energy ground state (GS) structures and transition structures (TS) and their computed enthalpies of activation (Δ*H*^‡^) for compounds **3**–**6**.

Calculations of isotope effects such as these depend mainly upon the predicted vibrational frequencies. The absolute energies of the ground and transition structures do not factor into the KIE, but it is still of interest to see if the barriers can be predicted with any accuracy. The computed B3LYP/6-31G(d,p) enthalpy of activation (Δ*H*^‡^, 308 K) for **3** is 18.3 kcal/mol, which is in reasonable agreement with the reported experimental value. The computed ground state and transition structures of cyclophane **3** are shown in Figure 3.

**Table 1:** KIEs for compounds **3**–**5** using scaled B3LYP/6-31G(d,p) harmonic frequencies via the Bigeleisen-Mayer or Δ*H*Δ*S* approaches.

*k*_H_/*k*_D_	3	4	5
Experimental	0.833 ± 0.040 ^a^	0.877 ± 0.024 ^b^	0.840 ± 0.050 ^c^
Bigeleisen-Mayer	0.832 ^a^	0.849 ^b^	0.825 ^c^
ZPE	0.829	0.822	0.820
EXC	0.999	1.027	1.001
MMI	1.005	1.005	1.005
ΔΔ*G*^‡^	0.833 ^a^	0.849 ^b^	0.825 ^c^
ΔΔ*H*^‡^	0.829	0.813	0.810
−TΔΔ*S*^‡^	1.005	1.044	1.018

^a^ 308 K; ^b^ 338 K; ^c^ 253 K.

Regarding the CKIE dissection in **3**-*d* (Table 1), it is clear that the effect is based almost entirely upon ZPE/enthalpy contributions. This is because the isotope effect is largely due to compression of a single C-H bond in the transition state (TS ν_CH_ = 3616 cm^−1^), which raises its frequency by some 400 cm^−1^ relative to the ground state (GS v_CH_ = 3206 cm^−1^). Isotopic substitution of D for H at this site in the transition structure leads to a greater decrease in ZPE relative to the ground state structures and speeds the interconversion process [6]. The important vibrational modes in this system are high-frequency and not likely to contribute any thermally excited terms. Indeed, the computed *S*_vib_, *H*_vib_, and EXC terms are insignificant. The calculations actually indicate that rotational differences (note that MMI = −TΔΔ*S*^‡^) are the likely source of the small entropic contribution. With such a dominant ZPE/enthalpic contribution, cyclophane **3** may be thought of as a molecule whose CKIE is purely steric in origin.

### 2.2. 1,1'-Binaphthyl-2,2'-d2(**4**-d_2_)

Another early example of a steric CKIE was reported in 1969 by Carter and Dahlgren in a fairly detailed study of the racemization process in 1,1-binaphthyl-2,2-*d*_2_ (**4**-*d*_2_, Figure 1) [22]. They determined *k*_H_/*k*_D_, = 0.877 ± 0.024 at 338 K, a value that became larger in magnitude (0.849 ± 0.024 at 302 K) at lower temperatures. The activation enthalpies and entropies for racemization in the unlabeled and labeled compounds were also measured (ΔΔ*H*^‡^ = 0.27 ± 0.14 kcal/mol, (TΔΔ*S*^‡^ = 0.54 ± 0.43 cal/mol K). The authors acknowledged the large uncertainties in these values when discussing their role in the CKIE, and were reluctant to conclude if the effect was purely enthalpic or steric in origin.

Our calculations find the CKIE in **4**-*d*_2_ (0.849, 338 K) consists of a dominant inverse ZPE/enthalpic contribution and a much smaller normal EXC/entropic contribution (Table 1). There is also a small mismatch between the ΔΔ*H*^‡^ and ZPE terms, reminiscent of the CKIE in dihydrodimethylphenanthrene **2**-*d*_6_. The computed ΔΔ*H*^‡^ (139 cal/mol) and ΔΔ*S*^‡^ (0.086 cal/mol K) terms fall within the uncertainty of the experimental values. The computed CKIE at 302 K is 0.828, a 2.1% reduction and consistent with the measured 2.8% reduction over the same temperature range. The computed enthalpy of activation (Δ*H*^‡^, 338 K) for **4** is 22.7 kcal/mol, which compares well with the experimental value (Δ*H*^‡^ = 21.49 ± 0.19 kcal/mol). The computed ground state and transition structures for **4** are shown in Figure 3.

### 2.3. 2,2'-Dibromo-4,4'-dicarboxybiphenyl (**5**-d_2_)

**Figure 4:**
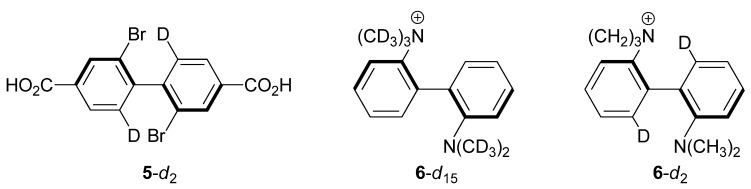
Substituted biphenyl compounds for which CKIEs have been measured.

Melander and Carter studied the steric isotope effect in the 6,6'-dideuterated form of 2,2'-dibromo-4,4-dicarboxybiphenyl (**5**-*d*_2_, Figure 4) and determined *k*_H_/*k*_D_, = 0.877 ± 0.024 at 253 K [23]. The enthalpy of activation in the unlabeled molecule is 18.5 kcal/mol [24]; our calculations predict Δ*H*^‡^ (253 K) = 17.3 kcal/mol (Figure 3). As with binaphthyl **4**-*d*_2_, our calculations slightly underestimate the experimental value and find the CKIE to consist of a dominant inverse ZPE/enthalpic component that is marginally offset by normal, though smaller, EXC/entropic contribution. Given the structural similarities between binaphthyl **4** and biphenyl **5**, it is perhaps not surprising to find similarities in the CKIE origins for these two systems. 

### 2.4. 2-(N,N,N-Trimethyl)-2'-(N,N-dimethyl)-diaminobiphenyl Cations 6-d_15_ and 6-d_2_

Isotopically labeled forms of diaminobiphenyl cation **6** (Figure 4) were investigated for steric isotope effects in two different laboratories in the late 1960s. Heitner and Leffek reported a surprisingly negligible CKIE at two temperatures (0.996 ± 0.007, 373 K; 1.02 ± 0.01, 393 K) in heavily labeled **6**-*d*_15_ (Table 2) [25]. The authors suggested that the small CKIE in **6**-*d*_15_ might be due to one of two things: (1) non-bonded interactions so large that deuteration does not make a difference; or (2) compensating enthalpic and entropic contributions, citing Mislow’s study of dihydro-dimethylphenanthrene **2**. They went on to say that “secondary deuterium isotope effects in biphenyl systems may be more complicated than simply steric effects,” noting that Melander and Carter’s dicarboxybiphenyl **5** lacked severe crowding but showed a large isotope effect per D—in contrast with the small CKIE in the severely crowded **6**-*d*_15_.

Spurred by Heitner and Leffek’s work, Carter and Dahlgren undertook a study of the more lightly labeled diaminobiphenyl **6**-*d*_2_ [26]. They were interested in this compound because they believed the 6 and 6' C-H/C-D bonds, and not the methyl groups, were in positions of maximal steric repulsion in the transition state. Their prediction was borne out by their measurement of large inverse CKIEs at several temperatures. At 371 K, for example, the CKIE was 0.847 ± 0.027 (Table 2). They also measured Δ*H*^‡^ for the unlabeled compound (27.37 ± 0.62 kcal/mol) and compared it with a measurement taken in a different laboratory (26.9 ± 0.62 kcal/mol [24]). Our B3LYP/6-31G(d,p) estimate for Δ*H*^‡^ (373 K) in the unlabeled compound is 26.2 kcal/mol. In discussing their results with reference to the Heitner/Leffek study, Carter and Dahlgren suggested that the **6**-*d*_15_ CKIE might be small simply because neither the quaternary nor the tertiary *N*-CH_3_/*N*-CD_3_ groups are in positions of steric repulsion. While this is true for the tertiary amine methyl groups (Figure 3), it is probably incorrect for the quaternary amine, as our calculations show that two of its C-H bonds are blue-shifted by some 150 cm^−1^ due to steric interactions with the 6'-H(D) group. This can be compared with the 6'-CH stretching frequency, which blue shifts in the transition structure by ca. 200 cm^−1^. Carter and Dahlgren were also skeptical of Heitner and Leffek’s arguments employing compensatory enthalpic and entropic effects, pointing to the difficulty in obtaining reliable ΔΔ*H*^‡^ and ΔΔ*S*^‡^ values from kinetic data measured over a limited temperature range. Our calculations of CKIEs in **6**-*d*_15_ and **6**-*d*_2_ are summarized in Table 2. The calculations for cation **6**-*d*_15_ predict a marginally normal isotope effect dominated by EXC/ΔΔ*S* terms. There is a small (ca. 2%) mismatch between the ZPE and ΔΔ*H*^‡^ terms, which suggests a small *H*_vib_ contribution is at play. On the other hand, the computed CKIE in **6**-*d*_2_ has a dominant and large enthalpic/ZPE contribution, although a small ZPE/ΔΔ*H*^‡^ mismatch remains.

**Table 2:** KIEs for compound **6** using scaled B3LYP/6-31G(d,p) harmonic frequencies via the Bigeleisen-Mayer or ΔHΔS approaches.

*k*_H_/*k*_D_	6-*d*_15_	6-*d*_15_	6-*d*_2_
Experimental	0.996 ± 0.007 ^a^	1.02 ± 0.01 ^b^	0.847 ± 0.027 ^c^
Bigeleisen-Mayer	1.041 ^a^	1.044 ^b^	0.829 ^c^
ZPE	0.966	0.968	0.798
EXC	1.089	1.090	1.034
MMI	0.989	0.989	1.005
ΔΔ*G*^‡^	1.041 ^a^	1.045 ^b^	0.828 ^c^
ΔΔ*H*^‡^	0.945	0.950	0.779
−TΔΔ*S*^‡^	1.101	1.100	1.063

^a^ 373 K; ^b^ 393 K; ^c^ 371 K.

These results support Carter and Dahlgren’s contention that deuterating the 6/6' positions in **6** would be critical for realizing a large steric isotope effect and provide evidence of compensatory enthalpic and entropic contributions in cation **6**-*d*_15_ as suggested by Heitner and Leffek. Our calculations suggest that entropy plays a dominant role in determining the CKIE in **6**-*d*_15_, which is consistent with other heavily deuterated substrates computed in our laboratories.

### 2.5. Compounds Structurally Similar to Mislow’s Doubly-Bridged Diketone **1**

**Figure 5:**
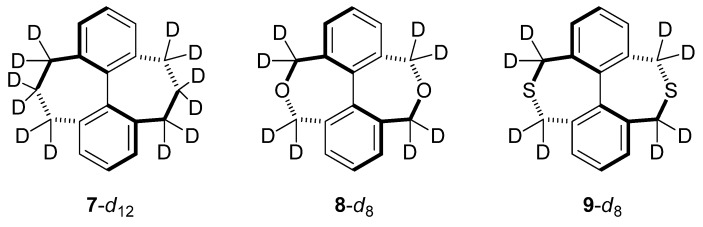
Analogs for *in silico* comparison with Mislow’s doubly-bridged ketone.

**Figure 6:**
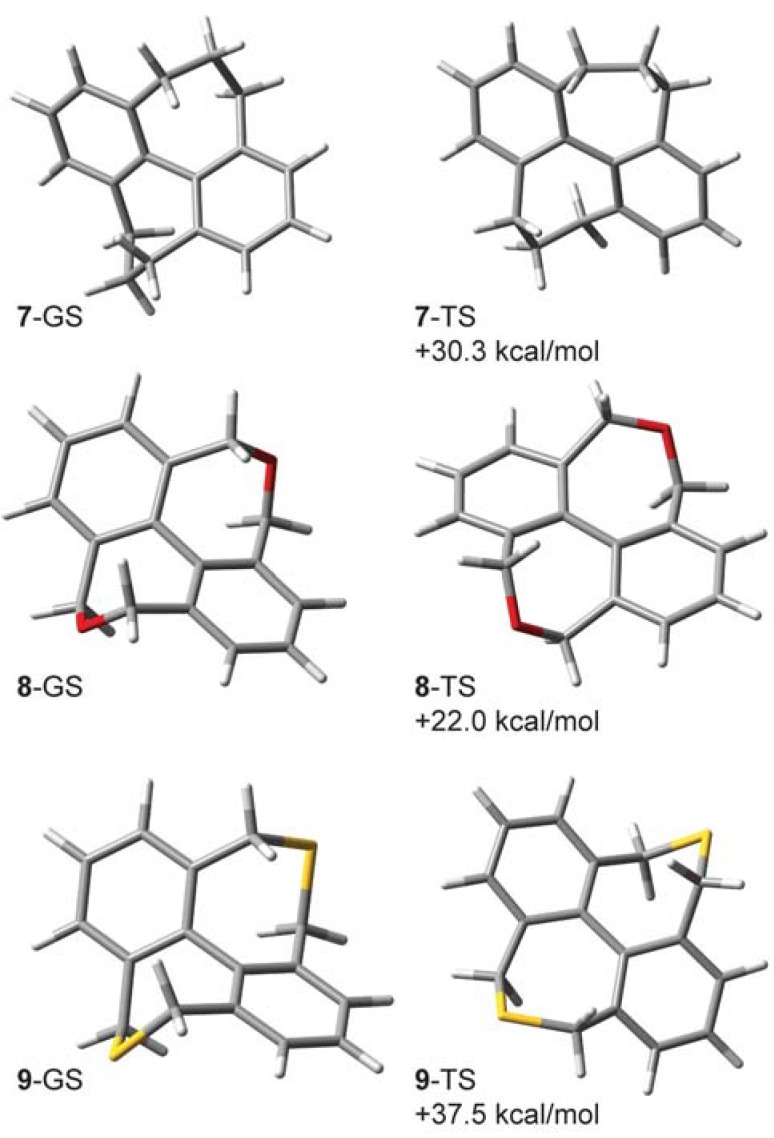
B3LYP/6-31G(d,p) lowest-energy ground state (GS) structures and transition structures (TS) and their computed enthalpies of activation (Δ*H*^‡^) for compounds **7**–**9**.

The origin of the normal CKIE in diketone **1**-*d*_8_ was reviewed earlier in this paper [5]. Here, we describe calculations of three analogs replacing the carbonyl groups with CH_2_, O, and S atoms (**7**, **8**, and **9**; Table 3; Figure 5, Figure 6). These are known compounds [10], but we are not aware of any prior studies involving the measurements of isotope effects in these systems.

**Table 3:** Computed CKIEs for compounds **1** and **7**–**9** using scaled B3LYP/6-31G(d,p) harmonic frequencies (368 K) via the Bigeleisen-Mayer or Δ*H*Δ*S* approaches.

*k*_H_/*k*_D_	1-*d*_8_	7-*d*_12_	8-*d*_8_	9-*d*_8_
Bigeleisen-Mayer	1.075	1.172	1.147	1.057
ZPE	1.026	1.022	1.069	0.989
EXC	1.050	1.148	1.073	1.069
MMI	0.998	0.998	0.999	1.000
ΔΔ*G*^‡^	1.075	1.171	1.146	1.058
ΔΔ*H*^‡^	0.973	0.935	1.005	0.931
−TΔΔ*S*^‡^	1.105	1.252	1.140	1.136

The computed enthalpies of activation (Δ*H*^‡^, 298 K) for **7**, **8**, and **9** are 30.3, 22.0 (experiment: 19.8 [10]), and 37.5 (experiment: 34.0 [10]) kcal/mol, respectively. These can be compared with the computed Δ*H*^‡^ for diketone **1**: 28.5 kcal/mol [5] (experiment: 30.4 kcal/mol [10]). To make a fair comparison with the computed and experimental CKIEs in **1**-*d*_8_, we report the Bigeleisen-Mayer and Δ*H*/Δ*S* dissections for compounds **7**–**9** as computed at 368 K.

It is interesting that the normal CKIE in the parent hydrocarbon **7**-*d*_12_ (1.171) and diether **8**-*d*_8_ (1.146) are each roughly twice the computed value in diketone **1**-*d*_8_ (1.075). The magnitude of the CKIE for dithioether **9**-*d*_8_ (1.057) and its dissected components more closely mirrors the behavior of diketone **1**-*d*_8_. The CKIEs in **7**-*d*_12_ and **8**-*d*_8_ are among the largest normal values that we know of, and a forthcoming paper will explore these effects in greater detail.

Like **1**-*d*_8_, compounds **7**–**9** have significant *H*_vib_ contributions and accordingly exhibit a large mismatch between ZPE and ΔΔ*H*^‡^; when expressed as ZPE − ΔΔ*H*^‡^ these values (**7-***d*_12_: 0.087, **8**-*d*_8_: 0.064, **9**-*d*_8_: 0.058) are greater than that calculated for **1**-*d*_8_ (0.053). The magnitude of the entropic term (1.252) in **7-***d*_12_ is uniquely large in this series of compounds and is likely a consequence of higher deuterium content. This large entropic factor is offset by a relatively large inverse enthalpic contribution (0.935). The CKIE in **8**-*d*_8_ is interesting because the enthalpic contribution (1.005) is nearly zero and the isotope effect is therefore governed mainly by vibrational entropy effects. The Bigeleisen-Mayer dissection for this compound suggests roughly equal and normal contributions from the ZPE and EXC terms.

### 2.6. Structures Related to Mislow’s Singly Bridged Dihydrodimethylphenanthrene **2**

**Figure 7:**
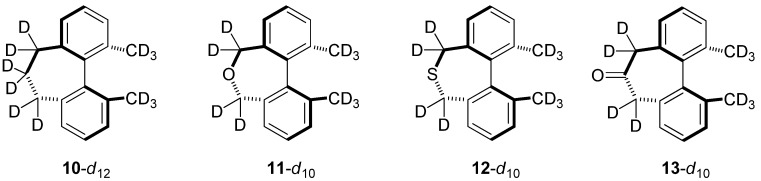
Analogs for *in silico* comparison with Mislow’s dihydrodimethylphenanthrene.

**Figure 8:**
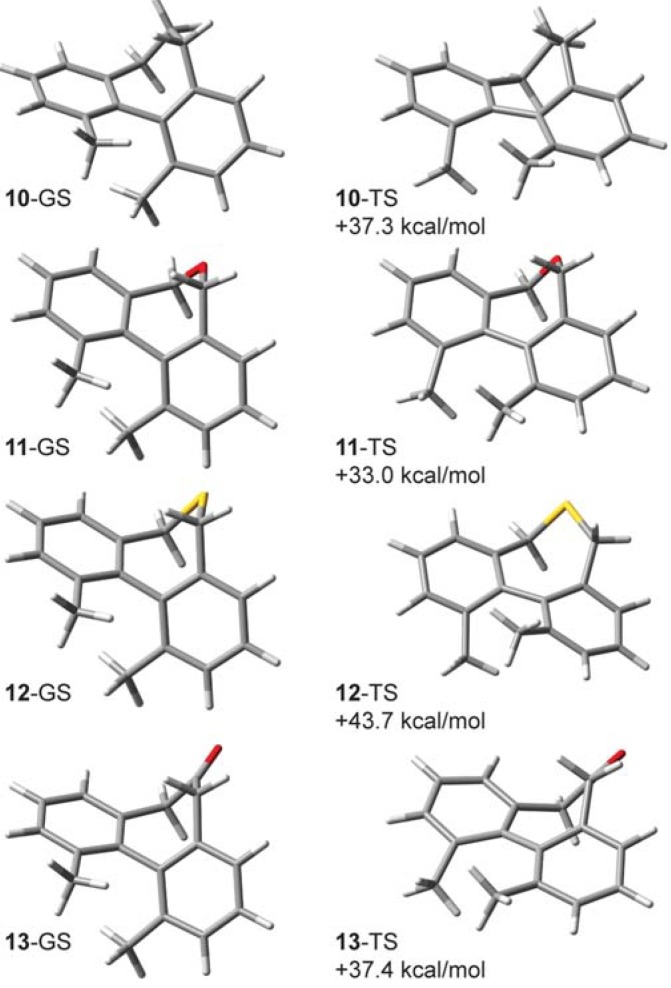
B3LYP/6-31G(d,p) lowest-energy ground state (GS) structures and transition structures (TS) and their computed enthalpies of activation (Δ*H*^‡^) for compounds **10**–**13**.

The final series of compounds in this study are structural analogs of Mislow’s dihydrodimethyl-phenanthrene **2** (**10**, **11**, **12**, and **13**; Table 4; Figure 7, Figure 8). As mentioned earlier, compound **2** represents an important milestone in conformational analysis because it is the first example of a steric isotope effect (albeit arising from antagonistic enthalpic/entropic contributions) [12,13]. The magnitude of these effects could be reproduced computationally [5], and the Δ*H*/Δ*S* and Bigeleisen-Mayer dissections for **2**-*d*_10_ are reproduced in Table 4 for purposes of comparison with analogs within this series.

**Table 4:** Computed CKIEs for compounds **2** and **10**–**13** using scaled B3LYP/6-31G(d,p) harmonic frequencies (315 K) via the Bigeleisen-Mayer or Δ*H*Δ*S* approaches.

*k*_H_/*k*_D_	2-*d*_10_	10-*d*_12_	11-*d*_10_	12-*d*_10_	13-*d*_10_
Bigeleisen-Mayer	0.846	0.869	0.868	0.959	0.867
ZPE	0.712	0.660	0.678	0.762	0.664
EXC	1.193	1.321	1.284	1.256	1.308
MMI	0.997	0.997	0.998	1.001	0.997
ΔΔ*G*^‡^	0.846	0.869	0.869	0.958	0.868
ΔΔ*H*^‡^	0.689	0.712	0.713	0.805	0.710
−TΔΔ*S*^‡^	1.227	1.221	1.219	1.191	1.219

Based upon experiment and theory, it is known that the CKIE in **2**-*d*_10_ is mainly due to deuteration at the methyl groups. As mentioned earlier, the computed CKIE for **2**-*d*_6_ is 0.888 and for **2**-*d*_4_ it is 0.952, which indicates that the bridging methylene groups do contribute an effect arising from antagonistic enthalpic and entropic terms [5]. A future study will explore the basis for this small but intriguing CKIE. In the present work, we will focus on analogs of the singly-bridged **2**-*d*_10_ in which the biphenyl system is annelated instead with a seven-membered ring. Like compounds **7**–**9**, the unlabeled forms of biphenyls **10**–**13** (Figure 7) have each been prepared, but little is known about their barriers to racemization. Our B3LYP/6-31G(d,p) estimates for their enthalpies of activation (Δ*H*^‡^, 298 K) can be compared with **1** (30.4 kcal/mol) and **2** (23.6 kcal/mol): **10** (37.3 kcal/mol), **11** (33.0 kcal/mol), **12** (43.7 kcal/mol), and **13** (37.4 kcal/mol).

In this series of compounds there is fair amount of similarity in the CKIEs and their origins for biphenyls **2**-*d*_10_, **10**-*d*_12_, **11**-*d*_10_, **12**-*d*_10_, and **13**-*d*_10_. Each has a net inverse isotope effect in the range of 0.84 to 0.87 arising from dominant and large inverse ZPE or ΔΔ*H*^‡^ terms.

Thiepin **12** appears to be an outlier with a smaller inverse effect (0.959) caused by a reduced ZPE/ ΔΔ*H*^‡^ contribution. The degree of mismatch between these terms (ZPE − ΔΔ*H*^‡^, **10-***d*_12_: −0.052, **11**-*d*_10_: −0.035, **12**-*d*_10_: −0.043, **13**-*d*_10_: −0.046) are greater and opposite in sign when compared with **2**-*d*_10_ (+0.023). With the partitioning behavior (Figure 2) in mind, this leads to some of the largest ZPE (0.66) and EXC (1.32) terms that we have encountered for systems computed at temperatures approximating experimental racemization conditions. 

### 2.7. On the Interplay and Relative Magnitudes of ΔΔH^‡^_ZPE_ and ΔΔH^‡^_vib_

Prior to this study, we had computed the thermal enthalpic contributions (ΔΔ*H*^‡^_thermal_ = ΔΔ*H*^‡^_ZPE_ + ΔΔ*H*^‡^_vib_) in only two examples (**1**-*d*_8_ and **2**-*d*_6_). We found it useful to express these contributions in terms of energy (kcal/mol), with a negative sign indicative of an inverse contribution to the isotope effect (Table 5). As described earlier, ΔΔ*H*^‡^_vib_ in diketone **1**-*d*_8_ is larger than ΔΔ*H*^‡^_ZPE_ and opposite in sign, which leads to the unusual situation where the Bigeleisen-Mayer ZPE and ΔΔ*H*^‡^_vib_ terms provide opposing normal and inverse CKIE contributions. The magnitude of either of these contributions in **1**-*d*_8_ is fairly small (ΔΔ*H*^‡^_vib_ = −0.038 kcal/mol ΔΔ*H*^‡^_ZPE_ = 0.018 kcal/mol) and when added together ΔΔ*H*^‡^_thermal_ contributes 0.973 to the CKIE. These values can be compared with those in **2**-*d*_6_, where two negative components of disparate magnitude (ΔΔ*H*^‡^_vib_ = −0.009 kcal/mol ΔΔ*H*^‡^_ZPE_ = −0.176 kcal/mol) combine such that ΔΔ*H*^‡^_thermal_ contributes a much larger and ZPE-dominated term (0.743) to the CKIE. 

**Table 5:** Frequency-dependent enthalpic^a^ KIE contributions (kcal/mol) for select systems.

Term	1-*d*_8_	7-*d*_12_	2-*d*_6_	2-*d*_10_	10-*d*_12_
ΔΔ*H*^‡^_vib_	−0.038	−0.065	−0.009	−0.020	+0.047
ΔΔ*H*^‡^_ZPE_	0.018	0.016	−0.176	−0.213	−0.260
ΔΔ*H*^‡^_thermal_	−0.020	−0.049	−0.185	−0.233	−0.213

^a^ ΔΔ*H*^‡^_thermal_ = ΔΔ*H*^‡^_ZPE_ + ΔΔ*H*^‡^_vib_.

Our calculations of doubly-bridged biphenyl **7**-*d*_12_, structurally related to **1**-*d*_8_, reveal an even larger relative ΔΔ*H*^‡^_thermal_ contribution (0.935) to the CKIE, arising from a larger ΔΔ*H*^‡^_vib_ term (−0.065 kcal/mol). Relative to **2**-*d*_6_, larger ΔΔ*H*^‡^_vib_ terms have also been computed in singly-bridged biphenyls **2**-*d*_10_ and **10**-*d*_12_. In each of these systems, the magnitude of ΔΔ*H*^‡^_vib_ is still very much smaller than ΔΔ*H*^‡^_ZPE_, and thus the enthalpic contribution remains ZPE-dominated. But it is the case that ΔΔ*H*^‡^_vib_ in **2**-*d*_10_ and **10**-*d*_12_ have opposite signs, and as alluded to in Section 2.5, the positive ΔΔ*H*^‡^_vib_ component in **10**-*d*_12_ reduces ΔΔ*H*^‡^_thermal_ such that ΔΔ*H*^‡^_ZPE_ or ZPE is greater than ΔΔ*H*^‡^_thermal_. The implication of these dissections is that, perhaps not surprisingly, virtually all possible sign combinations of ΔΔ*H*^‡^_vib_ and ΔΔ*H*^‡^_ZPE_ are possible and modulate the overall contribution of ΔΔ*H*^‡^_thermal._

A forthcoming paper will attempt to quantify the vibrational frequency dependence of ΔΔ*H*^‡^_vib_ for the purpose of understanding its contribution to isotope effects. A full treatment will require a quantitative linkage of vibrational frequencies with atomic coordinate displacement. Here, we simply remind the reader of the functional form and characteristic behavior of *H*_ZPE_ and *H*_vib_:

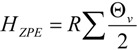
(1)

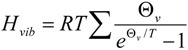
(2)
where 
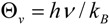


**Figure 9:**
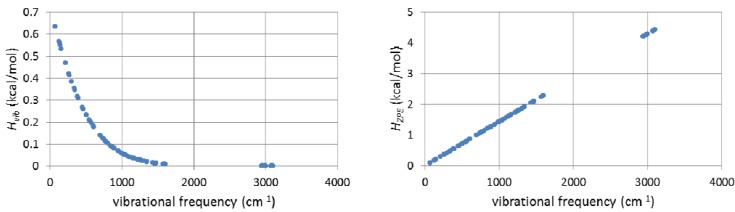
B3LYP/6-31G(d,p) *H*_vib_ (**left**) and *H*_ZPE_ (**right**) terms for the unlabeled ground state of biphenyl **7** computed at 368 K.

The frequency-dependent *H*_vib_ and *H*_ZPE_ terms for the ground state of unlabeled biphenyl **7** computed at 368 K are plotted in Figure 9. It needs to be remembered that the calculation of ΔΔ*H*^‡^_vib_ or ΔΔ*H*^‡^_ZPE_ involves evaluating the differences of these sums for labeled and unlabeled ground and transition structures. From the behavior of the two terms shown in Figure 9, it is apparent that only frequencies lower than 800 cm^−1^ make contributions greater than 0.1 kcal/mol to *H*_vib_ and, as one might expect from Equation 2, the maximal contribution is made by the lowest frequency motions. On the other hand, all frequencies make contributions greater than 0.1 kcal/mol to *H*_ZPE_, with the largest (4–4.5 kcal/mol) arising from the highest frequency bond stretching motions.

## 3. Computational Details

The calculations reported here utilized Gaussian 09 [27]. Our general strategy for locating ground state and transition structures involved starting with a reasonable ground state guess followed by relaxed scans of characteristic torsion angles. High-energy structures were then used as guesses for transition structure optimizations. In most cases, intrinsic reaction coordinate calculations (IRC) were used to correlate low- and intermediate-energy ground states and transition structures. B3LYP geometries were optimized using the opt=vtight, scf=(conver=10), and integral=(grid=ultrafine) keywords. IRC calculations utilized the default implementation. Δ*H*/Δ*S* KIEs were calculated using the default Gaussian 09 thermochemistry algorithm which accompanies harmonic frequency calculations, as adjusted for temperature and isotopic content. The Bigeleisen-Mayer terms were computed with a spreadsheet and the harmonic vibrational frequencies according to published protocols.

## 4. Conclusions

Our results provide additional examples of molecules with CKIEs governed by enthalpy-entropy interactions. This has provided an opportunity to examine additional systems with known isotope effects and lend insight to their origin. Of the thirteen compounds studied thus far, the following conclusions can be drawn: (1). The DFT B3LYP 6-31G(d,p) approach adequately reproduces the enthalpies of activation and CKIEs in medium-sized organic molecules. (2). Only Boekelheide’s cyclophane **3** comes closest to meeting the criteria for a ‘purely steric’ inverse CKIE because its dissected isotope effect is 99.5% determined by ZPE differences; this originates from a transition structure containing a highly compressed C-H(D) bond. (3). Vibrational entropy contributes to CKIEs in biphenyl derivatives; in lightly deuterated systems this can account for normal contributions of 1.02 (**5**-*d*_2_) to 1.06 (**6**-*d*_2_), whereas the –TΔΔ*S* contribution in heavily deuterated molecules can be as large as 1.25 (**7**-*d*_12_). In the systems studied thus far, the –TΔΔ*S* contribution is always normal (*k*_H_ > *k*_D_). (4). Non-bridged and singly-bridged biphenyl systems tend to have inverse CKIEs governed by ZPE-enthalpy contributions. Exceptions, such as **6**-*d*_15_, have large EXC or −TΔΔ*S*^‡^ contributions. (5). ZPE-enthalpy (or EXC-entropy) mismatches appear to be the rule, rather than the exception, in biphenyl-based CKIEs. For reasons not yet clear to us, doubly-bridged biphenyls appear to have particularly large mismatches; an additional example (**7**-*d*_12_) has been found in which the ZPE and ΔΔ*H*^‡^ terms actually predict opposite isotope effects. 6. Regarding mismatches in systems with inverse ZPE and ΔΔ*H*^‡^ terms (e.g., **2**-*d*_10_, **10**-*d*_12_, **11**-*d*_10_, **12**-*d*_10_, and **13**-*d*_10_), it is now clear that the ZPE term can in some cases be larger than ΔΔ*H*^‡^, which leads to inverse ZPE terms as large as 0.660 (**10**-*d*_12_) and normal EXC terms as large as 1.321 (**10**-*d*_12_).

Our studies underscore the necessity of properly comparing CKIE contributions in experimental and theoretical studies. The Bigeleisen-Mayer formalism is normally understood to have fairly clear correlations with enthalpic and entropic contributions, but there are instances in which *H*_vib_ can cause significant breakdowns in these correlations. We tend to favor the Δ*H*/Δ*S* partitioning over the Bigeleisen-Mayer approach because it represents what is measured experimentally. Furthermore, the analysis is conveniently done using default thermochemistry routines available within most computational packages. One current limitation of these routines is a lack of significant figures, which can be problematic for calculations of small isotope effects, such as those arising from ^13^C substitution [28]. For the methodology described here, we have shown that one can expect to find agreement equal to or better than 0.001 between the two approaches. Future work will further analyze the CKIEs in these and additional related systems, with the goal of understanding the origin of enthalpic and entropic contributions to isotope effects. 

## Supplementary Materials

Optimized ground and transition structure coordinate files can be accessed at: http://www.mdpi.com/1420-3049/18/2/2281/s1.
