# Supplementary material for: Enthalpy/Entropy Contributions to Conformational KIEs: Theoretical Predictions and Comparison with Experiment
Source: Molecules. 2013 Feb 18;18(2):2281–96. doi: 10.3390/molecules18022281 (PMC6269852; doi:10.3390/molecules18022281)
Supplement: Supplementary file 1 [file molecules-18-02281-s001.pdf]

Supplementary Material

Table of Contents

B3LYP/6-31G(d,p) optimized GS coordinates for cyclophane **3**……………………………………S2
B3LYP/6-31G(d,p) optimized TS coordinates for cyclophane **3**……………………………………S3
B3LYP/6-31G(d,p) optimized GS coordinates for binaphthyl **4**…………………………………… S4
B3LYP/6-31G(d,p) optimized TS coordinates for binaphthyl **4**…………………………………… S4
B3LYP/6-31G(d,p) optimized GS coordinates for diacid **5**………………………………………… S6
B3LYP/6-31G(d,p) optimized TS coordinates for diacid **5**…………………………………………S7
B3LYP/6-31G(d,p) optimized GS coordinates for diaminobiphenyl **6**………………………….…...S8
B3LYP/6-31G(d,p) optimized TS coordinates for diaminobiphenyl **6**………………………………S9
B3LYP/6-31G(d,p) optimized GS coordinates for biphenyl **7**………………………………........S10
B3LYP/6-31G(d,p) optimized TS coordinates for biphenyl **7**……………………………………S11
B3LYP/6-31G(d,p) optimized GS coordinates for biphenyl **8**…………………………………… S12
B3LYP/6-31G(d,p) optimized TS coordinates for biphenyl **8**……………………………………S13
B3LYP/6-31G(d,p) optimized GS coordinates for biphenyl **9**……………………………………S14
B3LYP/6-31G(d,p) optimized TS coordinates for biphenyl **9**……………………………………S15
B3LYP/6-31G(d,p) optimized GS coordinates for biphenyl **10**…………………………………… S16
B3LYP/6-31G(d,p) optimized TS coordinates for biphenyl **10**…………………………………… S17
B3LYP/6-31G(d,p) optimized GS coordinates for biphenyl **11**………………………….............. S18
B3LYP/6-31G(d,p) optimized TS coordinates for biphenyl **11**……………………………..........S19
B3LYP/6-31G(d,p) optimized GS coordinates for biphenyl **12**…………………………………… S20
B3LYP/6-31G(d,p) optimized TS coordinates for biphenyl **12**……………………………..........S21
B3LYP/6-31G(d,p) optimized GS coordinates for biphenyl **13**…………………………………… S22
B3LYP/6-31G(d,p) optimized TS coordinates for biphenyl **13**…………………………………… S23

%nprocshared=23

%mem=46GB
%chk=
freq rb3lyp/6-31g(d,p)

B3LYP/6-31G(d,p) optimized ground state geometry for cyclophane **3**

0 1

C	0.96586800	1.54929500	0.69608300
C	0.96586800	1.54929500	-0.69608300
C	1.46934900	0.45147800	-1.40626600
C	2.25673300	−0.46781200	−0.69879600
C	2.25673300	−0.46781200	0.69879600
C	1.46934900	0.45147800	1.40626600
H	0.42148900	2.32612300	1.22657200
H	0.42148900	2.32612300	−1.22657200
H	2.74103000	−1.28469600	−1.23005800
H	2.74103000	−1.28469600	1.23005800
C	0.83521700	0.08579100	−2.72967500
H	1.54199300	−0.38127800	−3.42566300
H	0.44412900	0.98529400	−3.21753300
C	0.83521700	0.08579100	2.72967500
H	1.54199300	−0.38127800	3.42566300
H	0.44412900	0.98529400	3.21753300
C	−0.35968500	−0.92727400	−2.48595300
H	−1.00175100	−0.92158600	−3.37445800
H	0.06483100	−1.93414400	−2.40603800
C	−0.35968500	−0.92727400	2.48595300
H	0.06483100	−1.93414400	2.40603800
H	−1.00175100	−0.92158600	3.37445800
C	−1.16320400	−0.62965700	1.22849600
C	−0.73031600	−1.14617400	0.00000000
C	−2.22355200	0.28599700	1.21332300
C	−1.16320400	−0.62965700	−1.22849600
H	0.06513900	−1.88269100	0.00000000
C	−2.77605600	0.70426300	0.00000000
H	−2.60412700	0.69043800	2.14848200
C	−2.22355200	0.28599700	−1.21332300
H	−3.61079500	1.40004000	0.00000000
H	−2.60412700	0.69043800	−2.14848200

%nprocshared=23

%mem=46GB
%chk=
freq rb3lyp/6-31g(d,p)

B3LYP/6-31G(d,p) optimized transition structure geometry for cyclophane **3**

0 1

C	2.03801300	0.53233500	−1.27461500
C	2.05356800	−0.85285000	−1.09582200
C	1.80437000	−1.38819000	0.18114800
C	2.03801300	−0.53233500	1.27461600
C	2.05356800	0.85285000	1.09582200
C	1.80437000	1.38819000	−0.18114800
H	1.98711600	0.93040100	−2.28521600
H	2.01832000	−1.49602300	−1.97199200
H	1.98711600	−0.93040100	2.28521700
H	2.01832000	1.49602300	1.97199300
C	0.93929300	−2.62894500	0.35889800
H	0.84603100	−2.84147700	1.42957000
H	1.38887900	−3.51824500	−0.10127800
C	0.93929300	2.62894500	−0.35889800
H	1.38887900	3.51824500	0.10127800
H	0.84603200	2.84147700	−1.42957000
C	−0.50956900	−2.50177800	−0.27275300
H	−0.38932000	−2.62532500	−1.35823300
H	−1.07648000	−3.38107800	0.05705500
C	−0.50956900	2.50177800	0.27275300
H	−0.38932000	2.62532500	1.35823300
H	−1.07648000	3.38107800	−0.05705500
C	−1.36124800	1.24107100	0.06377700
C	−0.73935000	0.00000000	0.00000000
C	−2.76313700	1.22673900	0.05193700
C	−1.36124800	−1.24107100	−0.06377800
H	0.31086000	0.00000000	0.00000000
C	−3.44101500	0.00000000	0.00000000
H	−3.33267700	2.15254600	0.09496500
C	−2.76313700	−1.22673900	−0.05193700
H	−4.52789600	0.00000000	0.00000000
H	−3.33267700	−2.15254600	−0.09496600

```
%nprocshared=23

%mem=46GB
%chk=
# freq rb3lyp/6-31g(d,p)

B3LYP/6-31G(d,p) optimized ground state geometry for binapthyl 4

0 1
 C   −1.28993900   2.47809500  −1.92747500
 C   −0.98941200   1.48399700  −1.02358600
 C    0.00039000   1.67900100  −0.02072300
 C    0.67184200   2.94612600   0.03648200
 C    0.33802100   3.94871700  −0.91388400
 C   −0.61864000   3.72285000  −1.87613200
 H   −2.04956300   2.30801700  −2.68485700
 H   −1.51155900   0.53455000  −1.06688000
 C    0.33802100   0.66787000   0.94049700
 C    1.64696600   3.17423600   1.04361400
 H    0.85408700   4.90407400  −0.86332700
 H   −0.86419600   4.49868900  −2.59538000
 C    1.94499700   2.19611200   1.96187900
 C    1.28768600   0.94490200   1.90720800
 H    2.14975600   4.13725400   1.07673200
 H    2.68878200   2.37518800   2.73270100
 H    1.53812400   0.17756000   2.63372700
 C   −0.33802100  −0.66787000   0.94049700
 C   −0.00039000  −1.67900100  −0.02072300
 C   −1.28768600  −0.94490200   1.90720800
 C    0.98941200  −1.48399700  −1.02358600
 C   −0.67184200  −2.94612600   0.03648200
 C   −1.94499700  −2.19611200   1.96187900
 H   −1.53812400  −0.17756000   2.63372700
 C    1.28993900  −2.47809500  −1.92747500
 H    1.51155900  −0.53455000  −1.06688000
 C   −0.33802100  −3.94871700  −0.91388400
 C   −1.64696600  −3.17423600   1.04361400
 H   −2.68878200  −2.37518800   2.73270100
 C    0.61864000  −3.72285000  −1.87613200
 H    2.04956300  −2.30801700  −2.68485700
 H   −0.85408700  −4.90407400  −0.86332700
 H   −2.14975600  −4.13725400   1.07673200
 H    0.86419600  −4.49868900  −2.59538000
```

%nprocshared=12

%chk=
freq rb3lyp/6-31g(d,p)

B3LYP/6-31G(d,p) optimized transition structure geometry for binapthyl **4**

0 1

C	−3.67523900	−1.88882200	−0.25239100
C	−2.36825300	−1.45611700	−0.34371500
C	−1.98490500	−0.10972500	−0.06801600
C	−3.07609100	0.81309900	0.12768500
C	−4.40465600	0.32955800	0.27536100
C	−4.70577400	−1.00178900	0.11916700
H	−3.91059800	−2.92083600	−0.49581300
H	−1.63629000	−2.15561600	−0.71739200
C	−0.61776200	0.41554400	−0.09428900
C	−2.85092300	2.21245700	0.07477700
H	−5.19117400	1.05351000	0.47187800
H	−5.72736600	−1.35538100	0.21997100
C	−1.60580700	2.67704400	−0.25940900
C	−0.51848000	1.78483400	−0.34356500
H	−3.68791700	2.88893200	0.22305300
H	−1.43168200	3.73652500	−0.42188900
H	0.43570100	2.20777800	−0.60843800
C	0.61776200	−0.41554400	0.09428900
C	1.98490500	0.10972500	0.06801600
C	0.51848000	−1.78483400	0.34356500
C	2.36825300	1.45611700	0.34371500
C	3.07609100	−0.81309900	−0.12768500
C	1.60580700	−2.67704400	0.25940900
H	−0.43570100	−2.20777800	0.60843800
C	3.67523900	1.88882200	0.25239100
H	1.63629000	2.15561600	0.71739200
C	4.40465600	−0.32955800	−0.27536100
C	2.85092300	−2.21245700	−0.07477700
H	1.43168200	−3.73652500	0.42188900
C	4.70577400	1.00178900	−0.11916700
H	3.91059800	2.92083600	0.49581300
H	5.19117400	−1.05351000	−0.47187800
H	3.68791700	−2.88893200	−0.22305300
H	5.72736600	1.35538100	−0.21997100

%nprocshared=6

%mem=12GB
%chk=
freq rb3lyp/6-31g(d,p)

B3LYP/6-31G(d,p) optimized ground state geometry for diacid **5**

0 1

C	−1.00853700	−2.72510200	1.80871400
C	−0.84673800	−1.34490600	1.74412000
C	−0.07971000	−0.74122900	0.73557800
C	0.54064500	−1.58121200	−0.20436400
C	0.38958000	−2.96110400	−0.15462800
C	−0.38958000	−3.53886400	0.85345900
H	−1.61079900	−3.17162600	2.59071200
H	−1.33020100	−0.70919300	2.47907800
H	0.87186200	−3.60119400	−0.88325400
C	0.07971000	0.74122900	0.73557800
C	0.84673800	1.34490600	1.74412000
C	−0.54064500	1.58121200	−0.20436400
C	1.00853700	2.72510200	1.80871400
H	1.33020100	0.70919300	2.47907800
C	−0.38958000	2.96110400	−0.15462800
C	0.38958000	3.53886400	0.85345900
H	1.61079900	3.17162600	2.59071200
H	−0.87186200	3.60119400	−0.88325400
C	0.51804300	5.02222400	0.85999100
O	−0.00430700	5.76573500	0.05507900
O	1.29215900	5.47890900	1.87486400
H	1.30793500	6.44597800	1.78278400
C	−0.51804300	−5.02222400	0.85999100
O	0.00430700	−5.76573500	0.05507900
O	−1.29215900	−5.47890900	1.87486400
H	−1.30793500	−6.44597800	1.78278400
Br	−1.63636700	0.84164500	−1.57903100
Br	1.63636700	−0.84164500	−1.57903100

%nprocshared=1

%mem=2GB
%chk=
freq rb3lyp/6-31g(d,p)

B3LYP/6-31G(d,p) optimized transition structure geometry for diacid **5**

0 1

C	−1.07155300	−0.00000100	−2.95776700
C	−1.07173400	−0.00000100	−1.57163400
C	0.08231100	0.00000000	−0.75211300
C	1.30175000	0.00000100	−1.50482600
C	1.31253100	0.00000000	−2.89882900
C	0.13808900	0.00000000	−3.64624300
H	−2.01255100	−0.00000200	−3.49500800
H	−2.03601900	−0.00000100	−1.10058500
H	2.25764400	0.00000100	−3.42661600
C	−0.08231100	0.00000000	0.75211300
C	1.07173400	0.00000100	1.57163400
C	−1.30175000	−0.00000100	1.50482600
C	1.07155300	0.00000100	2.95776700
H	2.03601900	0.00000100	1.10058500
C	−1.31253100	0.00000000	2.89882900
C	−0.13808900	0.00000000	3.64624300
H	2.01255100	0.00000200	3.49500800
H	−2.25764400	−0.00000100	3.42661600
C	−0.24623700	0.00000100	5.12938500
O	−1.29109900	0.00000000	5.74756600
O	0.96300500	0.00000200	5.74056100
H	0.78059900	0.00000200	6.69491100
C	0.24623700	−0.00000100	−5.12938500
O	1.29109900	0.00000000	−5.74756600
O	−0.96300500	−0.00000200	−5.74056100
H	−0.78059900	−0.00000200	−6.69491100
Br	−3.13350900	−0.00000200	0.86202100
Br	3.13350900	0.00000200	−0.86202100

%nprocshared=5

%mem=11GB
%chk=
freq rb3lyp/6-31g(d,p)

B3LYP/6-31G(d,p) optimized ground state geometry for diaminobiphenyl **6**

1 1

C	−2.06329900	2.54531700	−1.04620500
C	−0.82569000	1.91383800	−1.11286100
C	−0.58964400	0.62416500	−0.60062100
C	−1.70429400	−0.04443600	−0.03968900
C	−2.94703600	0.59266400	0.04451000
C	−3.12735800	1.88417000	−0.44574700
H	−2.18865600	3.54187300	−1.45618800
H	0.01207600	2.42555300	−1.57532800
H	−3.80517500	0.10738400	0.48429900
H	−4.10256400	2.35174100	−0.36444400
C	0.81443200	0.11104500	−0.79068100
C	1.15937700	−0.36621900	−2.06592200
C	1.81964800	0.20272900	0.21065300
C	2.44272800	−0.82843200	−2.35327700
H	0.39821300	−0.37924900	−2.84164000
C	3.10466100	−0.27634300	−0.09765600
C	3.41291700	−0.79357600	−1.35460200
H	2.67769300	−1.20500300	−3.34317400
H	3.88512500	−0.22372900	0.65222600
H	4.41966200	−1.14624600	−1.55547700
N	1.50395500	0.71888900	1.50380700
C	1.35697700	2.18198800	1.57504200
H	0.94789100	2.45047800	2.55322600
H	2.32083900	2.69961900	1.45015600
H	0.66963300	2.54252900	0.81190100
C	2.34641600	0.24241700	2.60345300
H	3.35581300	0.68343900	2.61195500
H	1.86647700	0.50966900	3.54947100
H	2.44855800	−0.84534100	2.56345300
C	−0.54259000	−1.70911400	1.45823500
H	0.42441800	−1.57671300	0.98802000
H	−0.67206400	−0.97036100	2.24589800
H	−0.64102200	−2.72298200	1.84710000
C	−1.35449800	−2.39239900	−0.77820000
H	−2.15508700	−2.24464300	−1.50247100
H	−0.39581800	−2.12103400	−1.21175000
H	−1.33593700	−3.42853700	−0.43739700
C	−2.91252700	−1.99056700	1.04310000
H	−2.75509600	−3.02686800	1.33826400
H	−3.14307600	−1.38968800	1.92138400
H	−3.72023400	−1.94272200	0.31589800
N	−1.62581000	−1.50069800	0.41868200

%nprocshared=23

%mem=46GB
%chk=
freq rb3lyp/6-31g(d,p)

B3LYP/6-31G(d,p) optimized transition structure geometry for diaminobiphenyl **6**

1 1

C	−0.69097200	3.05177100	−0.11058200
C	0.09102900	1.90513100	−0.11631600
C	−0.37513000	0.57227400	−0.00869200
C	−1.82276200	0.50465700	0.00690800
C	−2.60423900	1.66368900	0.06110600
C	−2.06303700	2.94223300	0.02391000
H	−0.21114000	4.02104900	−0.19907300
H	1.15892400	2.01593400	−0.23419800
H	−3.67834100	1.59921000	0.12228800
H	−2.71257700	3.80909800	0.07478700
C	0.70347000	−0.51205900	0.05354600
C	0.37892400	−1.87842700	0.23134600
C	2.12842300	−0.27165900	−0.07405000
C	1.27913700	−2.93800900	0.19591900
H	−0.63368400	−2.17667100	0.40544500
C	3.01663800	−1.35665400	−0.13898600
C	2.62324500	−2.68227100	−0.02603000
H	0.91576900	−3.95048000	0.33931200
H	4.07191600	−1.13698600	−0.24805200
H	3.35434300	−3.48262800	−0.07235400
N	2.73343400	1.02928400	−0.09285600
C	3.00846600	1.54466900	1.25655200
H	3.79487700	0.96924600	1.76877000
H	3.33173600	2.58754400	1.18528100
H	2.10276300	1.50680500	1.86748200
C	3.86652800	1.22390000	−0.99996000
H	3.63844400	0.79249400	−1.97662800
H	4.02423600	2.29922300	−1.12703100
H	4.81341800	0.79466400	−0.63635800
C	−2.59750600	−1.49330400	1.30015100
H	−1.59969500	−1.44571400	1.71864100
H	−3.27587700	−0.96526900	1.96863200
H	−2.92100600	−2.52841600	1.17958700
C	−2.24711500	−1.60890100	−1.27123900
H	−2.70010600	−1.13377100	−2.13998500
H	−1.17294700	−1.62341600	−1.39469100
H	−2.63241900	−2.62254000	−1.15441800
C	−4.14714600	−0.57003700	−0.28592000
H	−4.58629800	−1.56151400	−0.39835000
H	−4.60378200	−0.07473700	0.56675400
H	−4.29860800	0.00731400	−1.19516800
N	−2.65738500	−0.78392200	−0.05095100

%nprocshared=6

%mem=12GB
%chk=
freq rb3lyp/6-31g(d,p)

B3LYP/6-31G(d,p) optimized ground state geometry for biphenyl **7**

0 1

C	−2.85646400	−1.09465500	−0.50241500
C	−1.45824000	−1.11162600	−0.51024500
C	−0.74754700	0.00000000	0.00002000
C	−1.45821200	1.11162600	0.51032300
C	−2.85643600	1.09465600	0.50256800
C	−3.55668500	0.00000100	0.00009500
H	−3.39812000	−1.94848600	−0.90179000
H	−3.39807100	1.94848700	0.90197100
H	−4.64300500	0.00000100	0.00012400
C	0.74754800	0.00000000	−0.00002000
C	1.45821300	1.11163500	−0.51030400
C	1.45823900	−1.11163500	0.51022600
C	2.85643700	1.09466300	−0.50254900
C	2.85646300	−1.09466500	0.50239600
C	3.55668500	−0.00000100	−0.00009500
H	3.39807200	1.94850100	−0.90193700
H	3.39811900	−1.94850300	0.90175600
H	4.64300500	−0.00000100	−0.00012400
C	0.70684700	−2.29836300	1.07421600
H	−0.05158100	−1.93338800	1.77684200
H	1.39048000	−2.93023300	1.65108700
C	−0.70684800	−2.29834400	−1.07425600
H	0.05158000	−1.93335700	−1.77687600
H	−1.39048200	−2.93020400	−1.65113800
C	−0.70678900	2.29834400	1.07429400
H	0.05167600	1.93335700	1.77687300
H	−1.39039200	2.93020400	1.65121300
C	0.70679100	2.29836300	−1.07425400
H	1.39039400	2.93023300	−1.65116100
H	−0.05167500	1.93338800	−1.77684000
C	0.00000100	3.15394600	0.00002700
H	0.72601100	3.80780200	0.49846100
H	−0.72601000	3.80781000	−0.49839500
C	−0.00000100	−3.15394600	−0.00002800
H	−0.72598400	−3.80781000	0.49843400
H	0.72598300	−3.80780200	−0.49850000

%nprocshared=23

%mem=46GB
%chk=
freq rb3lyp/6-31g(d,p)

B3LYP/6-31G(d,p) optimized transition structure geometry for biphenyl **7**

0 1

C	−0.73674800	0.03859500	0.02683600
C	0.75242300	0.03304000	−0.04700300
C	−1.55967000	1.21633000	−0.04083400
C	1.54102300	1.22231600	−0.11695500
C	−1.44921600	−1.18842900	0.24243500
C	1.47977400	−1.19510300	−0.20982100
C	−2.90977700	1.07183800	−0.39310200
C	2.92523700	1.15702600	0.07771700
C	−2.80976200	−1.28191400	−0.06292400
C	2.86452000	−1.22001700	−0.01759300
C	−3.52679400	−0.17061200	−0.48878000
C	3.58902300	−0.05650900	0.21313600
C	−1.24092500	2.57241600	0.59608600
C	0.99496200	2.54729400	−0.60258100
C	−0.82692000	−2.35561000	0.99472500
C	0.85589500	−2.44687300	−0.80740500
H	−3.50654900	1.97119400	−0.52040000
H	3.49227300	2.08439200	0.07001100
H	−3.30979400	−2.23764900	0.07248500
H	3.38220800	−2.17182200	−0.10622100
H	−4.57075700	−0.25216200	−0.77618200
H	4.66104200	−0.09008600	0.38286700
H	−2.06618200	3.24173400	0.33444800
H	−1.35590700	2.39476500	1.67402400
H	1.84714700	3.17951100	−0.87389600
H	0.43480900	2.36809600	−1.53097900
H	−0.19107200	−1.94152700	1.78577200
H	−1.63106500	−2.90521900	1.49578700
H	0.19873100	−2.12835600	−1.62576400
H	1.65356900	−3.04361100	−1.26209400
C	0.09288700	3.30264000	0.35951500
H	0.61945800	3.45495100	1.30809500
H	−0.10186700	4.30144900	−0.04877600
C	0.02648300	−3.30055500	0.15011200
H	−0.59823600	−3.99590100	−0.42378700
H	0.66770400	−3.90499200	0.80322600

%nprocshared=4

%mem=8GB
%chk=
freq rb3lyp/6-31g(d,p)

B3LYP/6-31G(d,p) optimized ground state geometry for biphenyl **8**

0 1

C	−2.84845200	1.12573400	0.43142600
C	−1.45129900	1.13760700	0.44626900
C	−0.74304200	0.00000000	−0.00001800
C	−1.45127700	−1.13760700	−0.44633900
C	−2.84843100	−1.12573300	−0.43156400
C	−3.54859300	0.00000000	−0.00008600
H	−3.38988700	2.00389300	0.77325200
H	−3.38985100	−2.00389300	−0.77341600
H	−4.63450700	0.00000000	−0.00011200
C	0.74304200	0.00000000	0.00001800
C	1.45127700	−1.13764200	0.44625000
C	1.45129900	1.13764200	−0.44618000
C	2.84843100	−1.12576800	0.43147600
C	2.84845200	1.12576700	−0.43133800
C	3.54859300	0.00000000	0.00008600
H	3.38985000	−2.00395400	0.77326000
H	3.38988800	2.00395300	−0.77309600
H	4.63450700	−0.00000100	0.00011200
C	0.69189100	2.33269700	−0.98338600
H	−0.02229300	1.98709400	−1.74615600
H	1.38042900	3.03219000	−1.46506100
C	−0.69189000	2.33262000	0.98356700
H	0.02229400	1.98695900	1.74631000
H	−1.38042700	3.03207600	1.46529700
C	−0.69184300	−2.33262000	−0.98360000
H	0.02237900	−1.98695900	−1.74630800
H	−1.38035700	−3.03207600	−1.46536400
C	0.69184300	−2.33269700	0.98341900
H	1.38035700	−3.03219000	1.46512800
H	−0.02237800	−1.98709400	1.74615500
O	−0.00000100	−3.10307000	−0.00012000
O	0.00000000	3.10307000	0.00012000

%nprocshared=11

%mem=22GB
%chk=
freq rb3lyp/6-31g(d,p)

B3LYP/6-31G(d,p) optimized transition structure geometry for biphenyl **8**

0 1

C	−0.72872900	0.00445300	0.02547300
C	0.75067100	0.03905400	−0.02485500
C	−1.55166700	1.17742300	−0.01290100
C	1.48064000	1.25945700	−0.10087000
C	−1.41402100	−1.24070900	0.17549000
C	1.51440000	−1.16487000	−0.14387800
C	−2.91110700	1.04016300	−0.32298700
C	2.86865000	1.25825300	0.05993700
C	−2.78084100	−1.33310300	−0.10094100
C	2.90164800	−1.12937100	0.02566400
C	−3.51984700	−0.20620900	−0.44177700
C	3.58177700	0.07166200	0.20050700
C	−1.19978300	2.51595000	0.63163800
C	0.84157000	2.54228000	−0.58957200
C	−0.76080500	−2.42053800	0.88033300
C	0.92563400	−2.44037500	−0.72611500
H	−3.51498400	1.93859700	−0.41458800
H	3.39589200	2.20814800	0.03466500
H	−3.26697300	−2.30101800	−0.01452000
H	3.45590200	−2.06236500	−0.03053800
H	−4.57175600	−0.28505000	−0.69810000
H	4.65733200	0.08422900	0.34755200
H	−2.03794600	3.20166000	0.45653900
H	−1.17767900	2.31934100	1.71533400
H	1.63094900	3.25941600	−0.83033300
H	0.30150700	2.31736100	−1.52442800
H	−0.21691100	−2.02304200	1.75185700
H	−1.54437700	−3.08587000	1.25351800
H	0.34863800	−2.15239700	−1.62006100
H	1.74095100	−3.09083700	−1.05370100
O	−0.00955200	3.21235800	0.30994800
O	0.11084100	−3.23352100	0.11704200

```
%nprocshared=4

%mem=8GB
%chk=
# freq rb3lyp/6-31g(d,p)
```

B3LYP/6-31G(d,p) optimized ground state geometry for biphenyl **9**

```
0 1
 C    −1.06838300   −2.85764200    0.55772900
 C    −1.07877900   −1.45916600    0.57222600
 C     0.00000000   −0.74837800   −0.00002100
 C     1.07877800   −1.45913400   −0.57230800
 C     1.06838000   −2.85761100   −0.55788900
 C    −0.00000100   −3.55610100   −0.00010000
 H    −1.90267000   −3.39917900    0.99526500
 H     1.90266700   −3.39912400   −0.99545500
 H    −0.00000200   −4.64199600   −0.00013000
 C     0.00000000    0.74837800    0.00002100
 C     1.07882300    1.45913400    0.57222500
 C    −1.07882200    1.45916600   −0.57214400
 C     1.06842500    2.85761100    0.55780600
 C    −1.06842300    2.85764200   −0.55764700
 C     0.00000100    3.55610100    0.00009900
 H     1.90274700    3.39912300    0.99530800
 H    −1.90274400    3.39918000   −0.99512000
 H     0.00000200    4.64199700    0.00012900
 C    −2.21970200    0.71142200   −1.21233300
 H    −1.84781200   −0.10619400   −1.83492100
 H    −2.81774600    1.37393100   −1.84244300
 C    −2.21961000   −0.71142100    1.21250300
 H    −1.84767100    0.10619400    1.83506300
 H    −2.81760600   −1.37392900    1.84265900
 C     2.21960900   −0.71135400   −1.21254200
 H     1.84767100    0.10629700   −1.83505600
 H     2.81760500   −1.37382700   −1.84273600
 C     2.21970200    0.71135300    1.21237300
 H     2.81774700    1.37382700    1.84252000
 H     1.84781200   −0.10629700    1.83491600
 S    −3.42402800    0.00000100    0.00013100
 S     3.42402800   −0.00000200   −0.00013100
```

%nprocshared=12

%mem=24GB
%chk=
freq rb3lyp/6-31g(d,p)

B3LYP/6-31G(d,p) optimized transition structure geometry for biphenyl **9**

0 1

C	−0.05156300	−0.73647700	−0.00041900
C	−0.05171900	0.74875700	−0.09392900
C	−1.20473400	−1.59736800	−0.11498700
C	−1.23345700	1.54707400	−0.18227900
C	1.17727000	−1.42898100	0.25728500
C	1.17750200	1.47412100	−0.26112100
C	−1.00617300	−2.92213100	−0.53016200
C	−1.16687700	2.92817300	0.02356700
C	1.32997600	−2.77144600	−0.10636300
C	1.21158500	2.85616300	−0.04739700
C	0.26134800	−3.49386300	−0.61857000
C	0.04981500	3.58214200	0.18535300
C	−2.54946400	−1.40704900	0.58492700
C	−2.53031800	1.01008000	−0.72742900
C	2.26257600	−0.81813100	1.12060500
C	2.40622300	0.86624100	−0.90555600
H	−1.87984300	−3.54432500	−0.70231900
H	−2.09143000	3.49895500	0.00641700
H	2.29610600	−3.24514100	0.04490300
H	2.16741300	3.36759600	−0.12241400
H	0.38503500	−4.51792500	−0.95628300
H	0.08750100	4.65142600	0.36841500
H	−3.17908400	−2.26262200	0.33167700
H	−2.32961800	−1.48747600	1.65508700
H	−3.13935300	1.84508600	−1.08230000
H	−2.33031200	0.36390500	−1.58813600
H	1.81971800	−0.11099300	1.82587200
H	2.74291700	−1.61243500	1.69723000
H	2.10122000	0.12263200	−1.64643500
H	2.96039900	1.65068600	−1.42569300
S	−3.60829700	0.08587800	0.40734900
S	3.60188400	0.05517200	0.22345300

%nprocshared=4

%mem=8GB
%chk=
freq rb3lyp/6-31g(d,p)

B3LYP/6-31G(d,p) optimized ground state geometry for biphenyl **10**

0 1

C	2.91226500	−1.18440400	−0.52812000
C	1.51245000	−1.24419200	−0.59967000
C	0.75062800	−0.22172000	0.00912600
C	1.41524600	0.87464800	0.61474900
C	2.80963100	0.89789800	0.67072800
C	3.56100400	−0.13611200	0.11390700
H	3.49524800	−1.97285700	−0.99805000
H	3.30807900	1.73752900	1.14859000
H	4.64601500	−0.11203900	0.16199700
C	−0.75062800	−0.22171900	−0.00912500
C	−1.41524400	0.87464900	−0.61474900
C	−1.51245100	−1.24419100	0.59967000
C	−2.80963000	0.89790100	−0.67073000
C	−2.91226600	−1.18440100	0.52811800
C	−3.56100400	−0.13610900	−0.11390900
H	−3.30807600	1.73753300	−1.14859100
H	−3.49525100	−1.97285400	0.99804700
H	−4.64601500	−0.11203500	−0.16200100
C	0.88574700	−2.38701600	−1.36872300
H	0.85970600	−3.30839800	−0.77514400
H	−0.13760100	−2.16378100	−1.67588700
H	1.47183600	−2.60469900	−2.26720700
C	−0.88575200	−2.38701400	1.36872600
H	−0.85973300	−3.30840300	0.77515600
H	0.13760400	−2.16379000	1.67587100
H	−1.47183000	−2.60468000	2.26722100
C	0.60794700	2.04140100	1.13940200
H	−0.20682000	1.66934000	1.77035400
H	1.23457400	2.67674600	1.77413300
C	−0.60794400	2.04140200	−1.13940100
H	0.20682300	1.66934100	−1.77035300
H	−1.23457000	2.67674700	−1.77413200
C	0.00000200	2.89547400	0.00000100
H	−0.77041100	3.54866100	0.42744200
H	0.77041500	3.54866100	−0.42744000

%nprocshared=12

%mem=24GB
%chk=
freq rb3lyp/6-31g(d,p)

B3LYP/6-31G(d,p) optimized transition structure geometry for biphenyl **10**

0 1

C	0.01267500	3.01884200	−1.19575700
C	0.16581300	1.63977800	−1.40373300
C	0.12651800	0.74655800	−0.27822900
C	0.43645800	1.38228700	0.97832200
C	0.31717300	2.76491400	1.13784800
C	0.00000000	3.59061300	0.06765700
H	−0.02176800	3.66132100	−2.07244600
H	0.51834800	3.19242700	2.11684100
H	−0.12945400	4.66073000	0.19804800
C	−0.12651800	−0.74655800	−0.27822900
C	−0.43645800	−1.38228700	0.97832200
C	−0.16581300	−1.63977800	−1.40373300
C	−0.31717300	−2.76491400	1.13784800
C	−0.01267500	−3.01884200	−1.19575700
C	0.00000000	−3.59061300	0.06765700
H	−0.51834800	−3.19242700	2.11684100
H	0.02176800	−3.66132100	−2.07244600
H	0.12945400	−4.66073000	0.19804800
C	0.61146100	1.30198700	−2.81822800
H	1.22525100	0.40384200	−2.84445700
H	−0.19593700	1.19328100	−3.54657000
H	1.24131400	2.12478300	−3.16908700
C	−0.61146100	−1.30198700	−2.81822800
H	−1.22525100	−0.40384200	−2.84445700
H	0.19593700	−1.19328100	−3.54657000
H	−1.24131400	−2.12478300	−3.16908700
C	1.04302300	0.63743600	2.15815600
H	1.69584400	−0.15434600	1.77604000
H	1.68087000	1.33436600	2.71198300
C	−1.04302300	−0.63743600	2.15815600
H	−1.69584400	0.15434600	1.77604000
H	−1.68087000	−1.33436600	2.71198300
C	0.00000000	0.00000000	3.07598500
H	0.47244300	−0.74692900	3.72548100
H	−0.47244300	0.74692900	3.72548100

%nprocshared=3

%mem=6GB
%chk=
freq rb3lyp/6-31g(d,p)

B3LYP/6-31G(d,p) optimized ground state geometry for biphenyl **11**

0 1

C	2.92717900	−1.17007100	−0.47667600
C	1.52936900	−1.25522000	−0.55971600
C	0.74943200	−0.22549600	0.01335700
C	1.39731700	0.89982100	0.58035000
C	2.78963100	0.95532600	0.63884600
C	3.55866900	−0.09003000	0.13032600
H	3.52555000	−1.96415800	−0.91689000
H	3.27028100	1.82499600	1.07884100
H	4.64282100	−0.04930800	0.18138400
C	−0.74943200	−0.22549600	−0.01335700
C	−1.39731600	0.89982200	−0.58035000
C	−1.52936900	−1.25521900	0.55971600
C	−2.78963000	0.95532700	−0.63884700
C	−2.92717900	−1.17007000	0.47667600
C	−3.55866900	−0.09002800	−0.13032700
H	−3.27028000	1.82499700	−1.07884200
H	−3.52555100	−1.96415600	0.91688900
H	−4.64282100	−0.04930600	−0.18138600
C	0.92968300	−2.42321300	−1.31188400
H	0.92644300	−3.33739200	−0.70718800
H	−0.09842100	−2.22795800	−1.62212900
H	1.52011600	−2.63696500	−2.20838200
C	−0.92968500	−2.42321200	1.31188600
H	−0.92645200	−3.33739300	0.70719400
H	0.09842100	−2.22796000	1.62212500
H	−1.52011500	−2.63695700	2.20838800
C	0.56580800	2.06698500	1.06458700
H	−0.23448000	1.70984300	1.72825700
H	1.18371400	2.76881900	1.63101900
C	−0.56580600	2.06698500	−1.06458700
H	0.23448200	1.70984200	−1.72825700
H	−1.18371200	2.76881900	−1.63101900
O	0.00000100	2.84087200	0.00000000

%nprocshared=11

%mem=22GB
%chk=
freq rb3lyp/6-31g(d,p)

B3LYP/6-31G(d,p) optimized transition structure geometry for biphenyl **11**

0 1

C	0.01390600	3.03180600	−1.16267900
C	0.15464100	1.65512800	−1.39484300
C	0.12106300	0.74763700	−0.28134600
C	0.40671500	1.36787300	0.98666300
C	0.29362200	2.74735700	1.17071700
C	0.00000000	3.58905400	0.10768200
H	−0.02151600	3.68718700	−2.02953500
H	0.47082600	3.15435200	2.16232100
H	−0.12280600	4.65839100	0.24888300
C	−0.12106300	−0.74763700	−0.28134600
C	−0.40671500	−1.36787300	0.98666300
C	−0.15464100	−1.65512800	−1.39484300
C	−0.29362200	−2.74735700	1.17071700
C	−0.01390600	−3.03180600	−1.16267900
C	0.00000000	−3.58905400	0.10768200
H	−0.47082600	−3.15435200	2.16232100
H	0.02151600	−3.68718700	−2.02953500
H	0.12280600	−4.65839100	0.24888300
C	0.55540300	1.33733500	−2.82621700
H	1.20460200	0.46563600	−2.87867300
H	−0.27714800	1.19309300	−3.51882500
H	1.13451500	2.18448200	−3.20432500
C	−0.55540300	−1.33733500	−2.82621700
H	−1.20460200	−0.46563600	−2.87867300
H	0.27714800	−1.19309300	−3.51882500
H	−1.13451500	−2.18448200	−3.20432500
C	0.97732100	0.60167500	2.16780200
H	1.67464900	−0.16385500	1.79768700
H	1.54001200	1.29110100	2.80281900
C	−0.97732100	−0.60167500	2.16780200
H	−1.67464900	0.16385500	1.79768700
H	−1.54001200	−1.29110100	2.80281900
O	0.00000000	0.00000000	3.00324200

%nprocshared=4

%mem=8GB
%chk=
freq rb3lyp/6-31g(d,p)

B3LYP/6-31G(d,p) optimized ground state geometry for biphenyl **12**

0 1

C	2.90901400	−1.36169700	−0.57454200
C	1.50992300	−1.42498000	−0.63583400
C	0.75091600	−0.42410200	0.01079700
C	1.41693100	0.64351100	0.66023500
C	2.81292200	0.67604400	0.70112800
C	3.56030800	−0.33143400	0.09637200
H	3.49136300	−2.13176700	−1.07440200
H	3.31211600	1.49985400	1.20405600
H	4.64546100	−0.30393900	0.13299900
C	−0.75091600	−0.42410200	−0.01078900
C	−1.41693100	0.64349800	−0.66024700
C	−1.50992300	−1.42496800	0.63586000
C	−2.81292200	0.67603000	−0.70114000
C	−2.90901300	−1.36168800	0.57456700
C	−3.56030800	−0.33143700	−0.09636600
H	−3.31211700	1.49983100	−1.20408300
H	−3.49136200	−2.13174900	1.07444100
H	−4.64546100	−0.30394300	−0.13299200
C	0.87217900	−2.54941700	−1.42177100
H	0.80558300	−3.46889000	−0.82816200
H	−0.13783900	−2.29838400	−1.75165100
H	1.47238600	−2.78447300	−2.30604300
C	−0.87217800	−2.54939200	1.42181700
H	−0.80558500	−3.46887600	0.82822700
H	0.13784200	−2.29835300	1.75169000
H	−1.47238300	−2.78442900	2.30609600
C	0.61724400	1.76584400	1.26805600
H	−0.24426700	1.38893700	1.82370100
H	1.22842200	2.36584700	1.94638100
C	−0.61724500	1.76582100	−1.26808900
H	0.24426600	1.38890400	−1.82372700
H	−1.22842300	2.36581100	−1.94642500
S	0.00000000	2.97127800	−0.00002700

%nprocshared=23

%mem=46GB
%chk=
freq rb3lyp/6-31g(d,p)

B3LYP/6-31G(d,p) optimized transition structure geometry for biphenyl **12**

0 1

C	2.87855300	−1.54419900	−0.03788200
C	1.53491600	−1.47394500	0.35968300
C	0.74944000	−0.31924500	0.04206800
C	1.53096800	0.88707800	−0.14237300
C	2.85354600	0.76581200	−0.58716500
C	3.51024700	−0.46294800	−0.63242100
H	3.42943300	−2.46092000	0.15769300
H	3.40855600	1.67235000	−0.81125100
H	4.52910200	−0.53626000	−0.99986800
C	−0.73680200	−0.41329800	−0.06134200
C	−1.59647200	0.72855100	−0.16759700
C	−1.40465300	−1.67520200	−0.21581800
C	−2.97022800	0.60607300	0.05113400
C	−2.78574200	−1.76175100	0.00912600
C	−3.56574900	−0.63684900	0.23700600
H	−3.58194100	1.50374900	0.02117200
H	−3.25613900	−2.73979100	−0.05850600
H	−4.63005000	−0.72353300	0.43316000
C	1.07843900	−2.56382400	1.31653500
H	0.05698400	−2.42798400	1.66806900
H	1.17498000	−3.57035000	0.90005000
H	1.73564400	−2.52164100	2.19314000
C	−0.79000700	−2.92238400	−0.83222800
H	0.21035700	−2.75293500	−1.22831300
H	−0.74573100	−3.77013900	−0.14252400
H	−1.42963700	−3.22965700	−1.66656300
C	1.27783700	2.23861200	0.52605400
H	1.39762800	2.05364300	1.59906000
H	2.08871800	2.90903600	0.23349900
C	−1.13585400	2.03951400	−0.74821000
H	−0.47573000	1.85636900	−1.60196400
H	−2.00573000	2.58599800	−1.12045300
S	−0.27981700	3.20000600	0.35776100

%chk=

%mem=46GB
%nprocshared=23
freq rb3lyp/6-31g(d,p)

B3LYP/6-31G(d,p) optimized ground state geometry for biphenyl **13**

0 1

C	−0.01499000	0.74919100	−0.42292500
C	0.01499000	−0.74919100	−0.42292500
C	−0.64520000	1.48777200	−1.45140200
C	0.64520000	−1.48777200	−1.45140200
C	0.56351400	1.44103000	0.66988600
C	−0.56351400	−1.44103000	0.66988600
C	−0.61784300	2.88860100	−1.39874400
C	0.61784300	−2.88860100	−1.39874400
C	0.57320900	2.83757900	0.68920600
C	−0.57320900	−2.83757900	0.68920600
C	0.00000000	3.56387600	−0.35214900
C	0.00000000	−3.56387600	−0.35214900
C	−1.39511600	0.83318900	−2.59121700
C	1.39511600	−0.83318900	−2.59121700
C	1.10621600	0.68242200	1.86108000
C	−1.10621600	−0.68242200	1.86108000
C	0.00000000	0.00000000	2.67638200
O	0.00000000	0.00000000	3.88877200
H	−1.10529000	3.45201900	−2.19053100
H	1.10529000	−3.45201900	−2.19053100
H	1.02537400	3.35451500	1.53114700
H	−1.02537400	−3.35451500	1.53114700
H	0.01236600	4.64970500	−0.33304700
H	−0.01236600	−4.64970500	−0.33304700
H	−1.66789700	−0.19943900	−2.36671100
H	−2.31293300	1.38894100	−2.80706200
H	1.66789700	0.19943900	−2.36671100
H	2.31293300	−1.38894100	−2.80706200
H	1.78726600	−0.11561700	1.53469000
H	1.66003900	1.33541000	2.53858500
H	−1.78726600	0.11561700	1.53469000
H	−1.66003900	−1.33541000	2.53858500
H	0.80310800	−0.82678500	−3.51372100
H	−0.80310800	0.82678500	−3.51372100

%chk=

%mem=46GB
%nprocshared=23
freq rb3lyp/6-31g(d,p)

B3LYP/6-31G(d,p) optimized transition structure geometry for biphenyl **13**

```
0 1
 C    0.04169900    0.75500500   −0.49040600
 C   −0.04169900   −0.75500500   −0.49040600
 C   −0.03240900    1.64987200   −1.61521700
 C    0.03240900   −1.64987200   −1.61521700
 C    0.28324900    1.42567500    0.76217900
 C   −0.28324900   −1.42567500    0.76217900
 C   −0.34503800    3.00009200   −1.40416500
 C    0.34503800   −3.00009200   −1.40416500
 C    0.00000000    2.78537700    0.92703600
 C    0.00000000   −2.78537700    0.92703600
 C   −0.41782800    3.56671500   −0.13994100
 C    0.41782800   −3.56671500   −0.13994100
 C    0.43757800    1.36984500   −3.03420900
 C   −0.43757800   −1.36984500   −3.03420900
 C    0.96389200    0.77831200    1.95746200
 C   −0.96389200   −0.77831200    1.95746200
 C    0.00000000    0.00000000    2.82753000
 O    0.00000000    0.00000000    4.03948300
 H   −0.46046100    3.63431900   −2.27968100
 H    0.46046100   −3.63431900   −2.27968100
 H    0.14800200    3.23130500    1.90659500
 H   −0.14800200   −3.23130500    1.90659500
 H   −0.67355100    4.61326200   −0.00707600
 H    0.67355100   −4.61326200   −0.00707600
 H   −0.35715500    1.16002500   −3.75395000
 H    0.95441600    2.26557200   −3.39035900
 H    0.35715500   −1.16002500   −3.75395000
 H   −0.95441600   −2.26557200   −3.39035900
 H    1.72054000    0.06144800    1.61360400
 H    1.47000700    1.53425500    2.56133200
 H   −1.72054000   −0.06144800    1.61360400
 H   −1.47000700   −1.53425500    2.56133200
 H   −1.16021000   −0.55723600   −3.06742400
 H    1.16021000    0.55723600   −3.06742400
```
